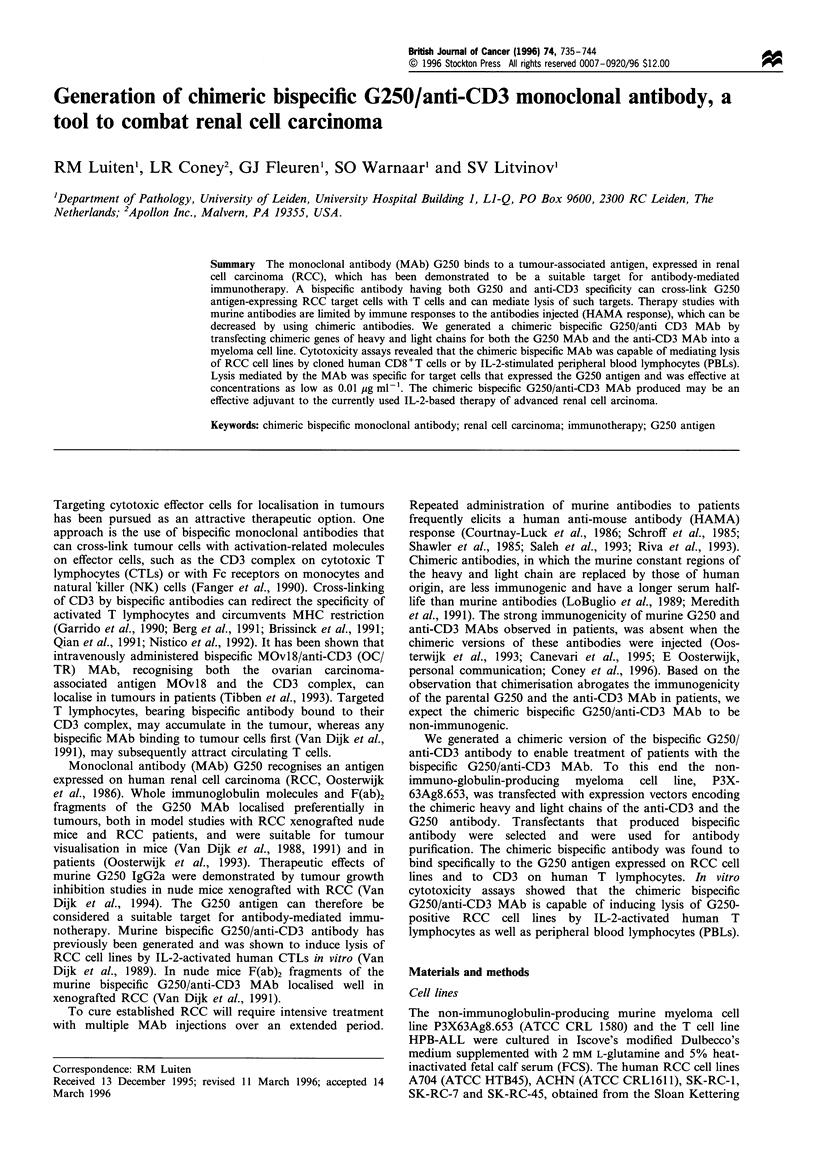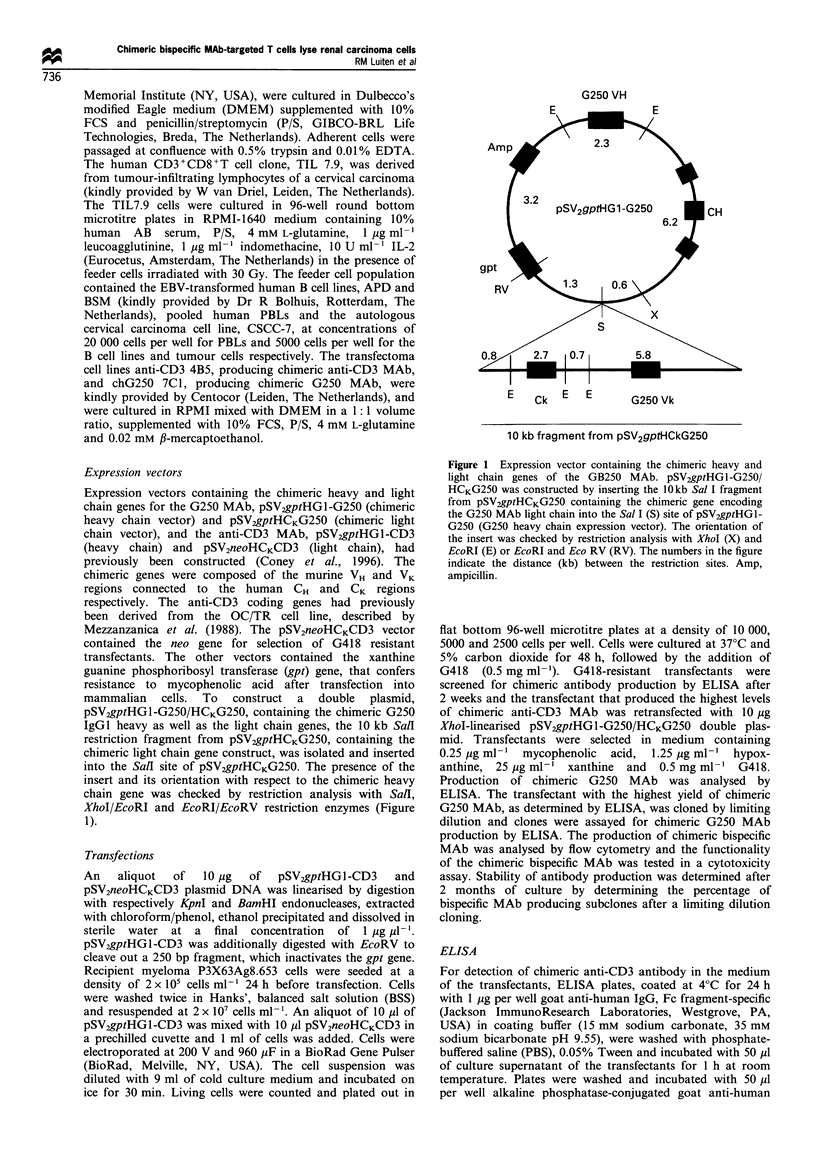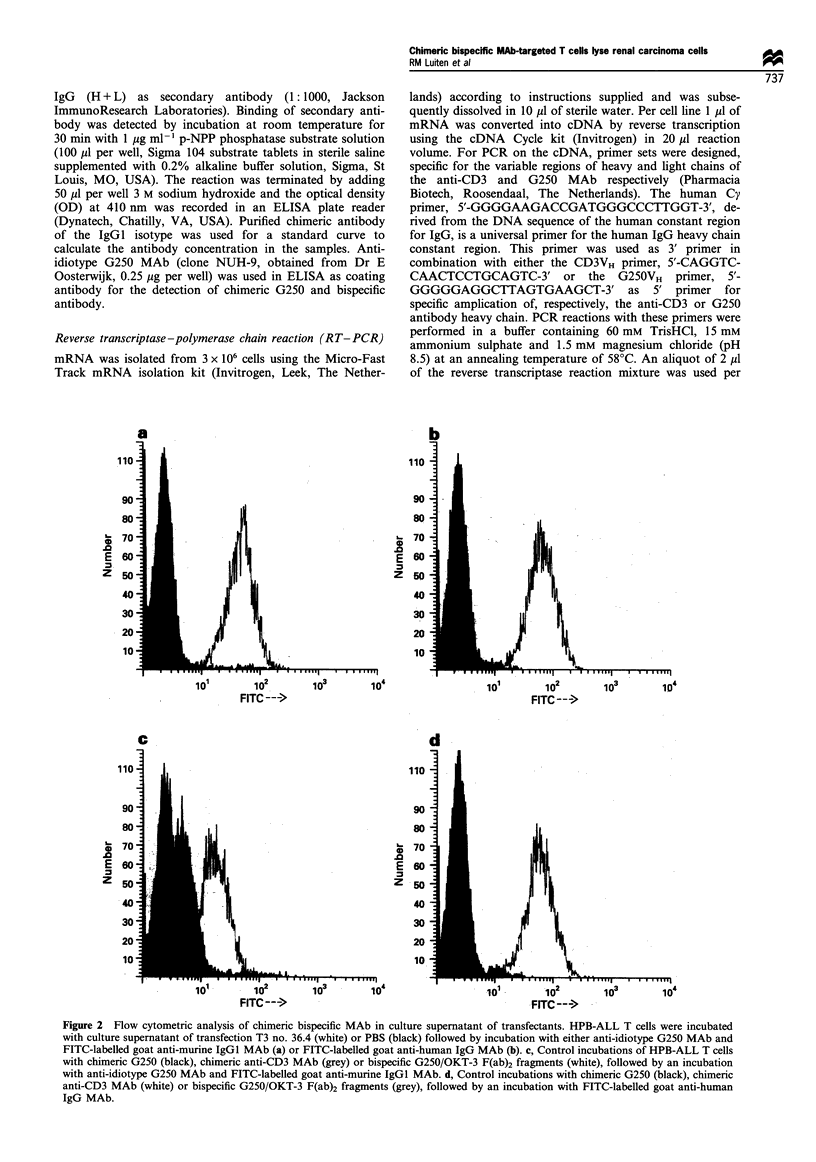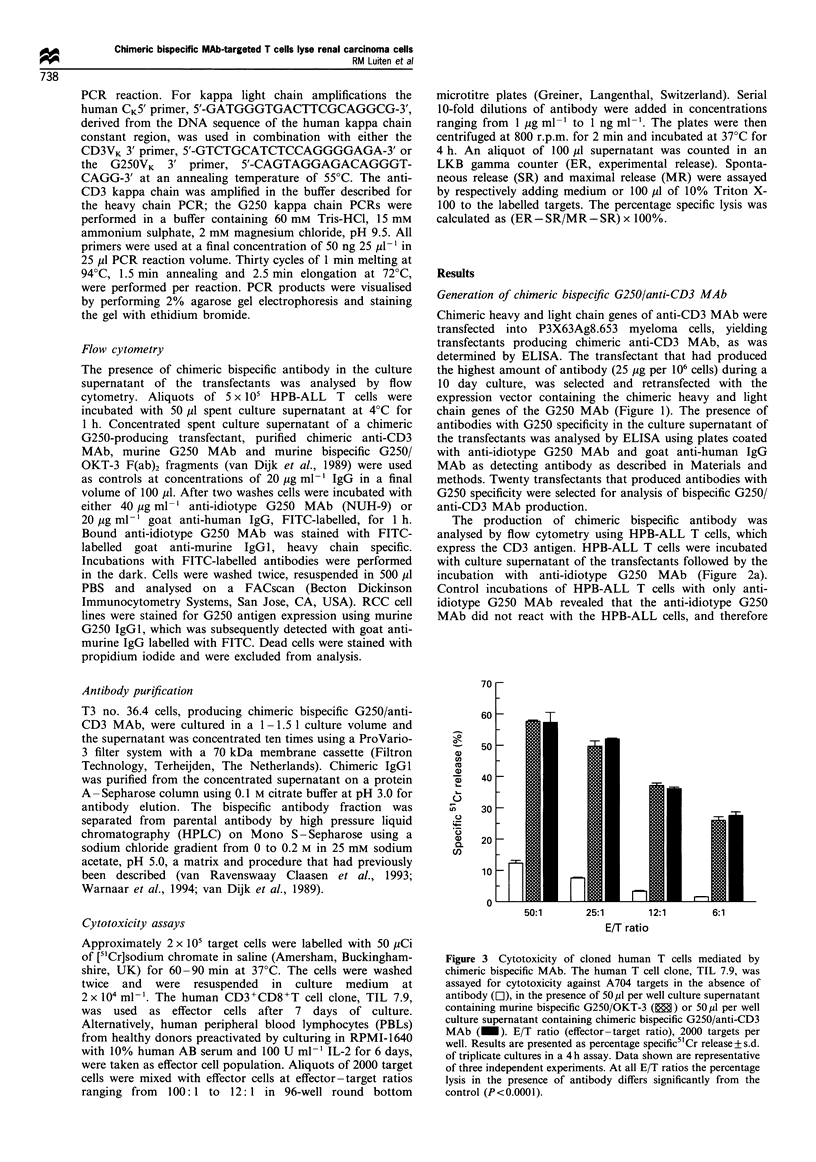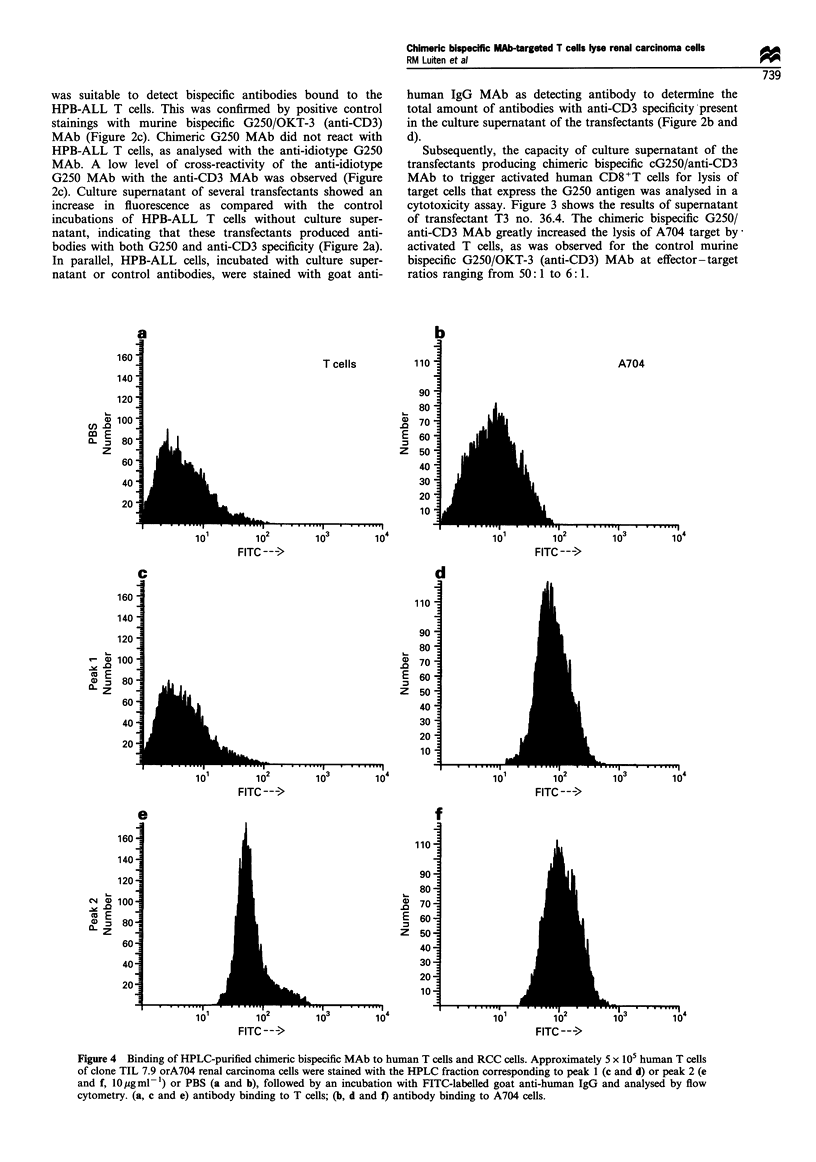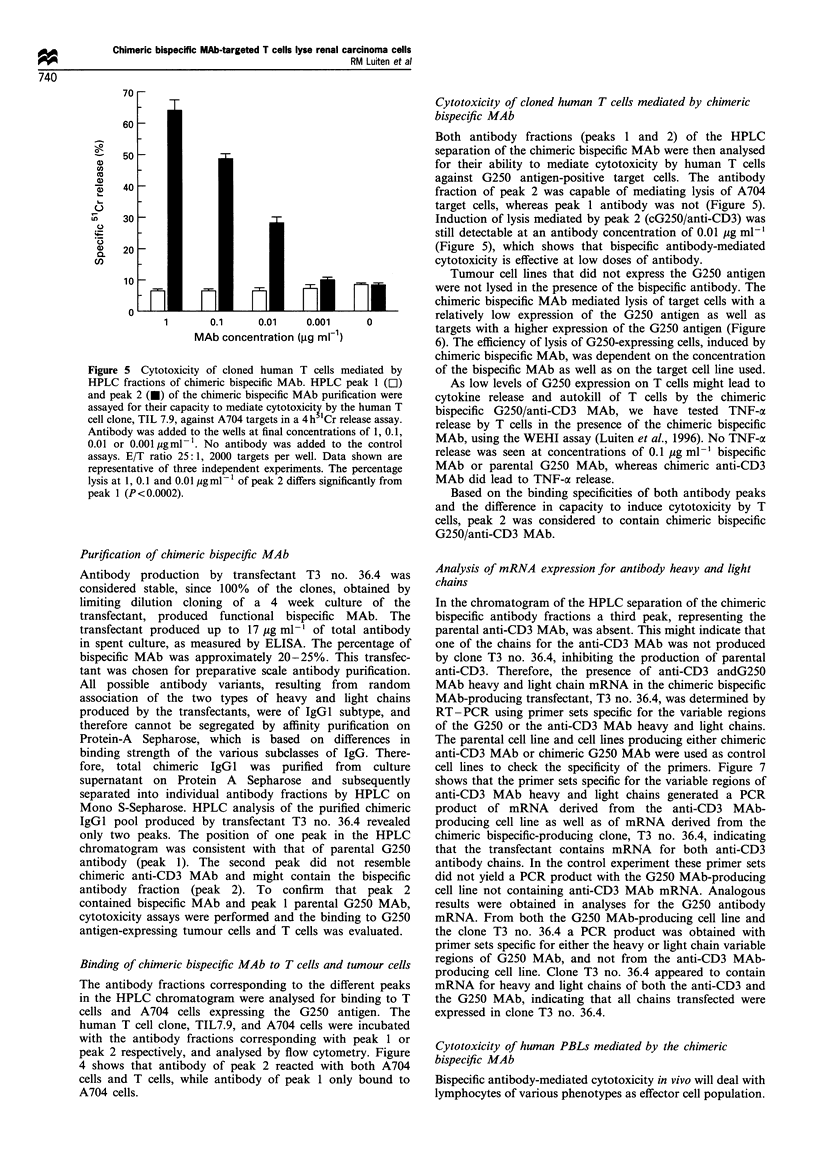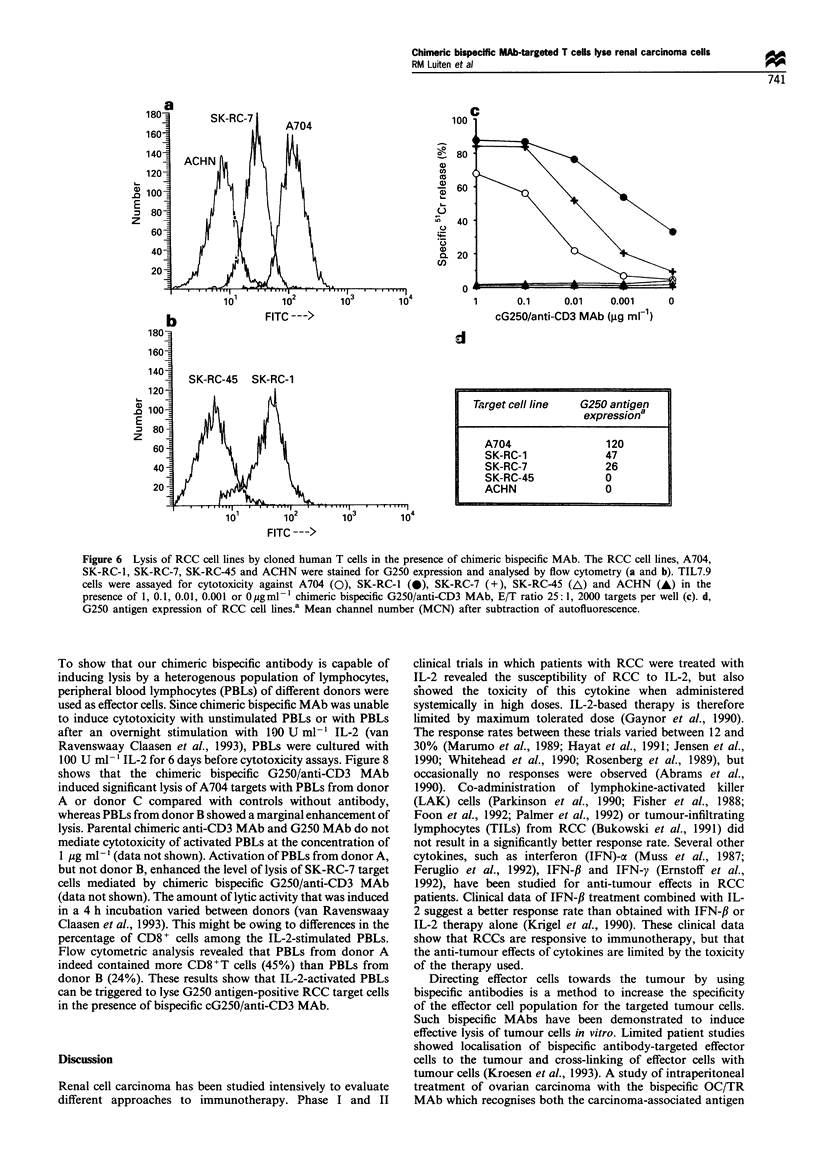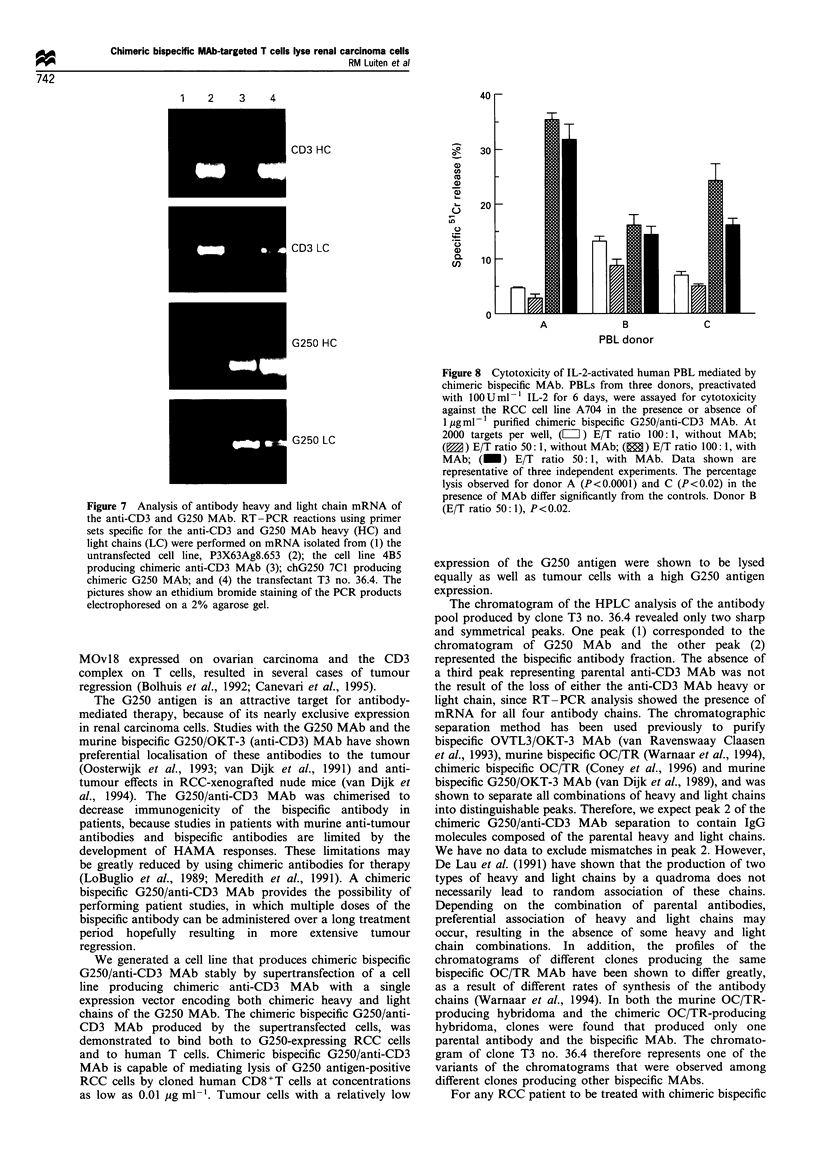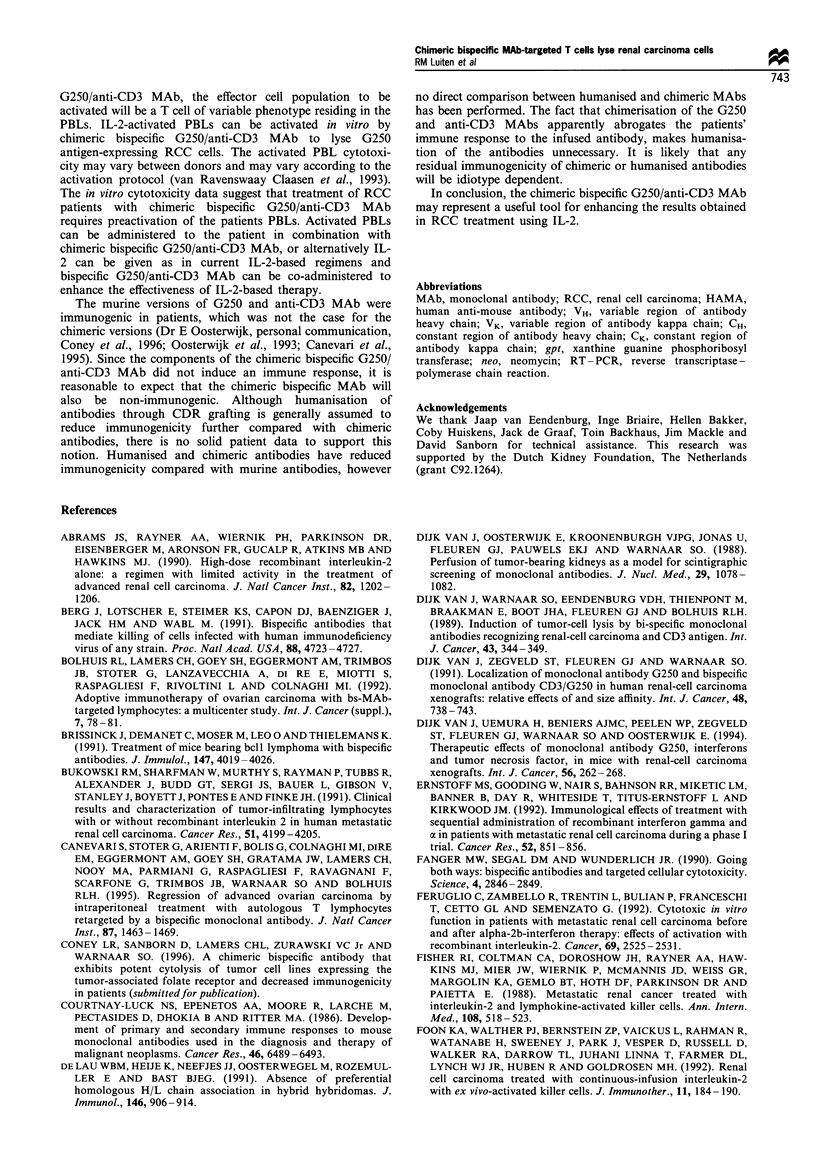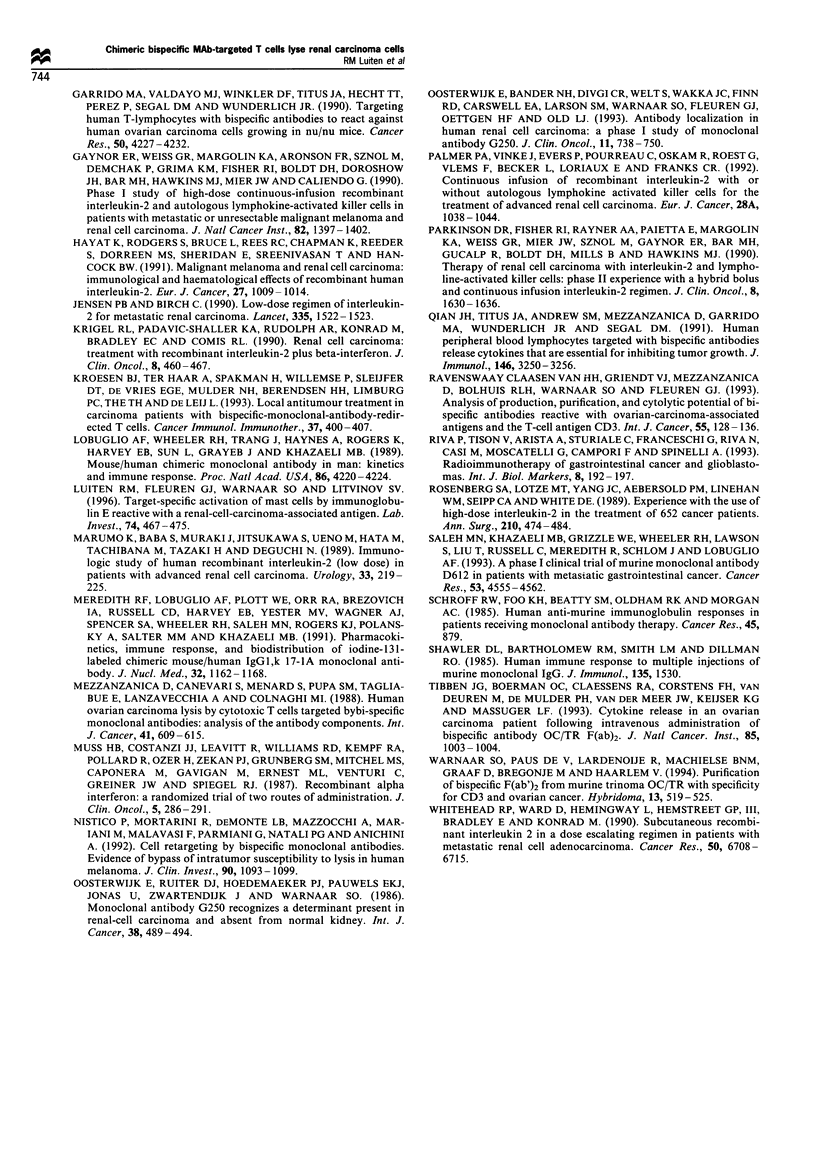# Generation of chimeric bispecific G250/anti-CD3 monoclonal antibody, a tool to combat renal cell carcinoma.

**DOI:** 10.1038/bjc.1996.430

**Published:** 1996-09

**Authors:** R. M. Luiten, L. R. Coney, G. J. Fleuren, S. O. Warnaar, S. V. Litvinov

**Affiliations:** Department of Pathology, University of Leiden, The Netherlands.

## Abstract

**Images:**


British Journal of Cancer (1996) 74, 735-744
© 1996 Stockton Press All rights reserved 0007-0920/96 $12.00

### Generation of chimeric bispecific G250/anti-CD3 monoclonal antibody, a tool to combat renal cell carcinoma

RM Luiten[1], LR Coney[2], GJ Fleuren[1], SO Warnaar[1] and SV Litvinov[1]

[1]Department of Pathology, University of Leiden, University Hospital Building 1, L1-Q, PO Box 9600, 2300 RC Leiden, The Netherlands; [2]Apollon Inc., Malvern, PA 19355, USA.

**Summary** The monoclonal antibody (MAb) G250 binds to a tumour-associated antigen, expressed in renal cell carcinoma (RCC), which has been demonstrated to be a suitable target for antibody-mediated immunotherapy. A bispecific antibody having both G250 and anti-CD3 specificity can cross-link G250 antigen-expressing RCC target cells with T cells and can mediate lysis of such targets. Therapy studies with murine antibodies are limited by immune responses to the antibodies injected (HAMA response), which can be decreased by using chimeric antibodies. We generated a chimeric bispecific G250/anti CD3 MAb by transfecting chimeric genes of heavy and light chains for both the G250 MAb and the anti-CD3 MAb into a myeloma cell line. Cytotoxicity assays revealed that the chimeric bispecific MAb was capable of mediating lysis of RCC cell lines by cloned human $CD8^{+}$T cells or by IL-2-stimulated peripheral blood lymphocytes (PBLs). Lysis mediated by the MAb was specific for target cells that expressed the G250 antigen and was effective at concentrations as low as 0.01 $\mu$g $ml^{-1}$. The chimeric bispecific G250/anti-CD3 MAb produced may be an effective adjuvant to the currently used IL-2-based therapy of advanced renal cell arcinoma.

**Keywords:** chimeric bispecific monoclonal antibody; renal cell carcinoma; immunotherapy; G250 antigen

Targeting cytotoxic effector cells for localisation in tumours has been pursued as an attractive therapeutic option. One approach is the use of bispecific monoclonal antibodies that can cross-link tumour cells with activation-related molecules on effector cells, such as the CD3 complex on cytotoxic T lymphocytes (CTLs) or with Fc receptors on monocytes and natural killer (NK) cells (Fanger *et al.*, 1990). Cross-linking of CD3 by bispecific antibodies can redirect the specificity of activated T lymphocytes and circumvents MHC restriction (Garrido *et al.*, 1990; Berg *et al.*, 1991; Brissinck *et al.*, 1991; Qian *et al.*, 1991; Nistico *et al.*, 1992). It has been shown that intravenously administered bispecific MOv18/anti-CD3 (OC/TR) MAb, recognising both the ovarian carcinoma-associated antigen MOv18 and the CD3 complex, can localise in tumours in patients (Tibben *et al.*, 1993). Targeted T lymphocytes, bearing bispecific antibody bound to their CD3 complex, may accumulate in the tumour, whereas any bispecific MAb binding to tumour cells first (Van Dijk *et al.*, 1991), may subsequently attract circulating T cells.

Monoclonal antibody (MAb) G250 recognises an antigen expressed on human renal cell carcinoma (RCC, Oosterwijk *et al.*, 1986). Whole immunoglobulin molecules and $F(ab)_2$ fragments of the G250 MAb localised preferentially in tumours, both in model studies with RCC xenografted nude mice and RCC patients, and were suitable for tumour visualisation in mice (Van Dijk *et al.*, 1988, 1991) and in patients (Oosterwijk *et al.*, 1993). Therapeutic effects of murine G250 IgG2a were demonstrated by tumour growth inhibition studies in nude mice xenografted with RCC (Van Dijk *et al.*, 1994). The G250 antigen can therefore be considered a suitable target for antibody-mediated immunotherapy. Murine bispecific G250/anti-CD3 antibody has previously been generated and was shown to induce lysis of RCC cell lines by IL-2-activated human CTLs *in vitro* (Van Dijk *et al.*, 1989). In nude mice $F(ab)_2$ fragments of the murine bispecific G250/anti-CD3 MAb localised well in xenografted RCC (Van Dijk *et al.*, 1991).

To cure established RCC will require intensive treatment with multiple MAb injections over an extended period. Repeated administration of murine antibodies to patients frequently elicits a human anti-mouse antibody (HAMA) response (Courtnay-Luck *et al.*, 1986; Schroff *et al.*, 1985; Shawler *et al.*, 1985; Saleh *et al.*, 1993; Riva *et al.*, 1993). Chimeric antibodies, in which the murine constant regions of the heavy and light chain are replaced by those of human origin, are less immunogenic and have a longer serum half-life than murine antibodies (LoBuglio *et al.*, 1989; Meredith *et al.*, 1991). The strong immunogenicity of murine G250 and anti-CD3 MAbs observed in patients, was absent when the chimeric versions of these antibodies were injected (Oosterwijk *et al.*, 1993; Canevari *et al.*, 1995; E Oosterwijk, personal communication; Coney *et al.*, 1996). Based on the observation that chimerisation abrogates the immunogenicity of the parental G250 and the anti-CD3 MAb in patients, we expect the chimeric bispecific G250/anti-CD3 MAb to be non-immunogenic.

We generated a chimeric version of the bispecific G250/anti-CD3 antibody to enable treatment of patients with the bispecific G250/anti-CD3 MAb. To this end the non-immuno-globulin-producing myeloma cell line, P3X-63Ag8.653, was transfected with expression vectors encoding the chimeric heavy and light chains of the anti-CD3 and the G250 antibody. Transfectants that produced bispecific antibody were selected and were used for antibody purification. The chimeric bispecific antibody was found to bind specifically to the G250 antigen expressed on RCC cell lines and to CD3 on human T lymphocytes. *In vitro* cytotoxicity assays showed that the chimeric bispecific G250/anti-CD3 MAb is capable of inducing lysis of G250-positive RCC cell lines by IL-2-activated human T lymphocytes as well as peripheral blood lymphocytes (PBLs).

### Materials and methods

### *Cell lines*

The non-immunoglobulin-producing murine myeloma cell line P3X63Ag8.653 (ATCC CRL 1580) and the T cell line HPB-ALL were cultured in Iscove's modified Dulbecco's medium supplemented with 2 mM L-glutamine and 5% heat-inactivated fetal calf serum (FCS). The human RCC cell lines A704 (ATCC HTB45), ACHN (ATCC CRL1611), SK-RC-1, SK-RC-7 and SK-RC-45, obtained from the Sloan Kettering

Correspondence: RM Luiten
Received 13 December 1995; revised 11 March 1996; accepted 14 March 1996

Memorial Institute (NY, USA), were cultured in Dulbecco's modified Eagle medium (DMEM) supplemented with 10% FCS and penicillin/streptomycin (P/S, GIBCO-BRL Life Technologies, Breda, The Netherlands). Adherent cells were passaged at confluence with 0.5% trypsin and 0.01% EDTA. The human $CD3^+CD8^+$T cell clone, TIL 7.9, was derived from tumour-infiltrating lymphocytes of a cervical carcinoma (kindly provided by W van Driel, Leiden, The Netherlands). The TIL7.9 cells were cultured in 96-well round bottom microtitre plates in RPMI-1640 medium containing 10% human AB serum, P/S, 4 mM L-glutamine, 1 $\mu$g ml$^{-1}$ leucoagglutinine, 1 $\mu$g ml$^{-1}$ indomethacine, 10 U ml$^{-1}$ IL-2 (Eurocetus, Amsterdam, The Netherlands) in the presence of feeder cells irradiated with 30 Gy. The feeder cell population contained the EBV-transformed human B cell lines, APD and BSM (kindly provided by Dr R Bolhuis, Rotterdam, The Netherlands), pooled human PBLs and the autologous cervical carcinoma cell line, CSCC-7, at concentrations of 20 000 cells per well for PBLs and 5000 cells per well for the B cell lines and tumour cells respectively. The transfectoma cell lines anti-CD3 4B5, producing chimeric anti-CD3 MAb, and chG250 7C1, producing chimeric G250 MAb, were kindly provided by Centocor (Leiden, The Netherlands), and were cultured in RPMI mixed with DMEM in a 1 : 1 volume ratio, supplemented with 10% FCS, P/S, 4 mM L-glutamine and 0.02 mM $\beta$-mercaptoethanol.

**Figure 1** Expression vector containing the chimeric heavy and light chain genes of the GB250 MAb. pSV$_2$*gpt*HG1-G250/HC$_K$G250 was constructed by inserting the 10 kb *Sal* I fragment from pSV$_2$*gpt*HC$_K$G250 containing the chimeric gene encoding the G250 MAb light chain into the *Sal* I (S) site of pSV$_2$*gpt*HG1-G250 (G250 heavy chain expression vector). The orientation of the insert was checked by restriction analysis with *Xho*I (X) and *Eco*RI (E) or *Eco*RI and *Eco* RV (RV). The numbers in the figure indicate the distance (kb) between the restriction sites. Amp, ampicillin.

*Expression vectors*

Expression vectors containing the chimeric heavy and light chain genes for the G250 MAb, pSV$_2$*gpt*HG1-G250 (chimeric heavy chain vector) and pSV$_2$*gpt*HC$_K$G250 (chimeric light chain vector), and the anti-CD3 MAb, pSV$_2$*gpt*HG1-CD3 (heavy chain) and pSV$_2$*neo*HC$_K$CD3 (light chain), had previously been constructed (Coney *et al.*, 1996). The chimeric genes were composed of the murine $V_H$ and $V_K$ regions connected to the human $C_H$ and $C_K$ regions respectively. The anti-CD3 coding genes had previously been derived from the OC/TR cell line, described by Mezzanzanica *et al.* (1988). The pSV$_2$*neo*HC$_K$CD3 vector contained the *neo* gene for selection of G418 resistant transfectants. The other vectors contained the xanthine guanine phosphoribosyl transferase (*gpt*) gene, that confers resistance to mycophenolic acid after transfection into mammalian cells. To construct a double plasmid, pSV$_2$*gpt*HG1-G250/HC$_K$G250, containing the chimeric G250 IgG1 heavy as well as the light chain genes, the 10 kb *Sal*I restriction fragment from pSV$_2$*gpt*HC$_K$G250, containing the chimeric light chain gene construct, was isolated and inserted into the *Sal*I site of pSV$_2$*gpt*HC$_K$G250. The presence of the insert and its orientation with respect to the chimeric heavy chain gene was checked by restriction analysis with *Sal*I, *Xho*I/*Eco*RI and *Eco*RI/*Eco*RV restriction enzymes (Figure 1).

*Transfections*

An aliquot of 10 $\mu$g of pSV$_2$*gpt*HG1-CD3 and pSV$_2$*neo*HC$_K$CD3 plasmid DNA was linearised by digestion with respectively *Kpn*I and *Bam*HI endonucleases, extracted with chloroform/phenol, ethanol precipitated and dissolved in sterile water at a final concentration of 1 $\mu$g $\mu$l$^{-1}$. pSV$_2$*gpt*HG1-CD3 was additionally digested with *Eco*RV to cleave out a 250 bp fragment, which inactivates the *gpt* gene. Recipient myeloma P3X63Ag8.653 cells were seeded at a density of $2 \times 10^5$ cells ml$^{-1}$ 24 h before transfection. Cells were washed twice in Hanks', balanced salt solution (BSS) and resuspended at $2 \times 10^7$ cells ml$^{-1}$. An aliquot of 10 $\mu$l of pSV$_2$*gpt*HG1-CD3 was mixed with 10 $\mu$l pSV$_2$*neo*HC$_K$CD3 in a prechilled cuvette and 1 ml of cells was added. Cells were electroporated at 200 V and 960 $\mu$F in a BioRad Gene Pulser (BioRad, Melville, NY, USA). The cell suspension was diluted with 9 ml of cold culture medium and incubated on ice for 30 min. Living cells were counted and plated out in flat bottom 96-well microtitre plates at a density of 10 000, 5000 and 2500 cells per well. Cells were cultured at 37°C and 5% carbon dioxide for 48 h, followed by the addition of G418 (0.5 mg ml$^{-1}$). G418-resistant transfectants were screened for chimeric antibody production by ELISA after 2 weeks and the transfectant that produced the highest levels of chimeric anti-CD3 MAb was retransfected with 10 $\mu$g *Xho*I-linearised pSV$_2$*gpt*HG1-G250/HC$_K$G250 double plasmid. Transfectants were selected in medium containing 0.25 $\mu$g ml$^{-1}$ mycophenolic acid, 1.25 $\mu$g ml$^{-1}$ hypoxanthine, 25 $\mu$g ml$^{-1}$ xanthine and 0.5 mg ml$^{-1}$ G418. Production of chimeric G250 MAb was analysed by ELISA. The transfectant with the highest yield of chimeric G250 MAb, as determined by ELISA, was cloned by limiting dilution and clones were assayed for chimeric G250 MAb production by ELISA. The production of chimeric bispecific MAb was analysed by flow cytometry and the functionality of the chimeric bispecific MAb was tested in a cytotoxicity assay. Stability of antibody production was determined after 2 months of culture by determining the percentage of bispecific MAb producing subclones after a limiting dilution cloning.

*ELISA*

For detection of chimeric anti-CD3 antibody in the medium of the transfectants, ELISA plates, coated at 4°C for 24 h with 1 $\mu$g per well goat anti-human IgG, Fc fragment-specific (Jackson ImmunoResearch Laboratories, Westgrove, PA, USA) in coating buffer (15 mM sodium carbonate, 35 mM sodium bicarbonate pH 9.55), were washed with phosphate-buffered saline (PBS), 0.05% Tween and incubated with 50 $\mu$l of culture supernatant of the transfectants for 1 h at room temperature. Plates were washed and incubated with 50 $\mu$l per well alkaline phosphatase-conjugated goat anti-human

IgG (H+L) as secondary antibody (1:1000, Jackson ImmunoResearch Laboratories). Binding of secondary antibody was detected by incubation at room temperature for 30 min with 1 $\mu$g ml$^{-1}$ p-NPP phosphatase substrate solution (100 $\mu$l per well, Sigma 104 substrate tablets in sterile saline supplemented with 0.2% alkaline buffer solution, Sigma, St Louis, MO, USA). The reaction was terminated by adding 50 $\mu$l per well 3 M sodium hydroxide and the optical density (OD) at 410 nm was recorded in an ELISA plate reader (Dynatech, Chatilly, VA, USA). Purified chimeric antibody of the IgG1 isotype was used for a standard curve to calculate the antibody concentration in the samples. Anti-idiotype G250 MAb (clone NUH-9, obtained from Dr E Oosterwijk, 0.25 $\mu$g per well) was used in ELISA as coating antibody for the detection of chimeric G250 and bispecific antibody.

*Reverse transcriptase–polymerase chain reaction (RT–PCR)*

mRNA was isolated from $3 \times 10^6$ cells using the Micro-Fast Track mRNA isolation kit (Invitrogen, Leek, The Netherlands) according to instructions supplied and was subsequently dissolved in 10 $\mu$l of sterile water. Per cell line 1 $\mu$l of mRNA was converted into cDNA by reverse transcription using the cDNA Cycle kit (Invitrogen) in 20 $\mu$l reaction volume. For PCR on the cDNA, primer sets were designed, specific for the variable regions of heavy and light chains of the anti-CD3 and G250 MAb respectively (Pharmacia Biotech, Roosendaal, The Netherlands). The human C$\gamma$ primer, 5′-GGGGAAGACCGATGGGCCCTTGGT-3′, derived from the DNA sequence of the human constant region for IgG, is a universal primer for the human IgG heavy chain constant region. This primer was used as 3′ primer in combination with either the CD3V$_H$ primer, 5′-CAGGTCCAACTCCTGCAGTC-3′ or the G250V$_H$ primer, 5′-GGGGGAGGCTTAGTGAAGCT-3′ as 5′ primer for specific amplication of, respectively, the anti-CD3 or G250 antibody heavy chain. PCR reactions with these primers were performed in a buffer containing 60 mM TrisHCl, 15 mM ammonium sulphate and 1.5 mM magnesium chloride (pH 8.5) at an annealing temperature of 58°C. An aliquot of 2 $\mu$l of the reverse transcriptase reaction mixture was used per

**Figure 2** Flow cytometric analysis of chimeric bispecific MAb in culture supernatant of transfectants. HPB-ALL T cells were incubated with culture supernatant of transfection T3 no. 36.4 (white) or PBS (black) followed by incubation with either anti-idiotype G250 MAb and FITC-labelled goat anti-murine IgG1 MAb (**a**) or FITC-labelled goat anti-human IgG MAb (**b**). **c**, Control incubations of HPB-ALL T cells with chimeric G250 (black), chimeric anti-CD3 MAb (grey) or bispecific G250/OKT-3 F(ab)$_2$ fragments (white), followed by an incubation with anti-idiotype G250 MAb and FITC-labelled goat anti-murine IgG1 MAb. **d**, Control incubations with chimeric G250 (black), chimeric anti-CD3 MAb (white) or bispecific G250/OKT-3 F(ab)$_2$ fragments (grey), followed by an incubation with FITC-labelled goat anti-human IgG MAb.

PCR reaction. For kappa light chain amplifications the human $C_K5'$ primer, 5′-GATGGGTGACTTCGCAGGCG-3′, derived from the DNA sequence of the human kappa chain constant region, was used in combination with either the $CD3V_K$ 3′ primer, 5′-GTCTGCATCTCCAGGGGAGA-3′ or the $G250V_K$ 3′ primer, 5′-CAGTAGGAGACAGGGT-CAGG-3′ at an annealing temperature of 55°C. The anti-CD3 kappa chain was amplified in the buffer described for the heavy chain PCR; the G250 kappa chain PCRs were performed in a buffer containing 60 mM Tris-HCl, 15 mM ammonium sulphate, 2 mM magnesium chloride, pH 9.5. All primers were used at a final concentration of 50 ng 25 $\mu l^{-1}$ in 25 $\mu$l PCR reaction volume. Thirty cycles of 1 min melting at 94°C, 1.5 min annealing and 2.5 min elongation at 72°C, were performed per reaction. PCR products were visualised by performing 2% agarose gel electrophoresis and staining the gel with ethidium bromide.

### *Flow cytometry*

The presence of chimeric bispecific antibody in the culture supernatant of the transfectants was analysed by flow cytometry. Aliquots of $5 \times 10^5$ HPB-ALL T cells were incubated with 50 $\mu$l spent culture supernatant at 4°C for 1 h. Concentrated spent culture supernatant of a chimeric G250-producing transfectant, purified chimeric anti-CD3 MAb, murine G250 MAb and murine bispecific G250/OKT-3 $F(ab)_2$ fragments (van Dijk *et al.*, 1989) were used as controls at concentrations of 20 $\mu g\ ml^{-1}$ IgG in a final volume of 100 $\mu$l. After two washes cells were incubated with either 40 $\mu g\ ml^{-1}$ anti-idiotype G250 MAb (NUH-9) or 20 $\mu g\ ml^{-1}$ goat anti-human IgG, FITC-labelled, for 1 h. Bound anti-idiotype G250 MAb was stained with FITC-labelled goat anti-murine IgG1, heavy chain specific. Incubations with FITC-labelled antibodies were performed in the dark. Cells were washed twice, resuspended in 500 $\mu$l PBS and analysed on a FACscan (Becton Dickinson Immunocytometry Systems, San Jose, CA, USA). RCC cell lines were stained for G250 antigen expression using murine G250 IgG1, which was subsequently detected with goat anti-murine IgG labelled with FITC. Dead cells were stained with propidium iodide and were excluded from analysis.

### *Antibody purification*

T3 no. 36.4 cells, producing chimeric bispecific G250/anti-CD3 MAb, were cultured in a 1–1.5 l culture volume and the supernatant was concentrated ten times using a ProVario-3 filter system with a 70 kDa membrane cassette (Filtron Technology, Terheijden, The Netherlands). Chimeric IgG1 was purified from the concentrated supernatant on a protein A–Sepharose column using 0.1 M citrate buffer at pH 3.0 for antibody elution. The bispecific antibody fraction was separated from parental antibody by high pressure liquid chromatography (HPLC) on Mono S–Sepharose using a sodium chloride gradient from 0 to 0.2 M in 25 mM sodium acetate, pH 5.0, a matrix and procedure that had previously been described (van Ravenswaay Claasen *et al.*, 1993; Warnaar *et al.*, 1994; van Dijk *et al.*, 1989).

### *Cytotoxicity assays*

Approximately $2 \times 10^5$ target cells were labelled with 50 $\mu$Ci of [$^{51}$Cr]sodium chromate in saline (Amersham, Buckinghamshire, UK) for 60–90 min at 37°C. The cells were washed twice and were resuspended in culture medium at $2 \times 10^4\ ml^{-1}$. The human $CD3^+CD8^+$T cell clone, TIL 7.9, was used as effector cells after 7 days of culture. Alternatively, human peripheral blood lymphocytes (PBLs) from healthy donors preactivated by culturing in RPMI-1640 with 10% human AB serum and 100 U $ml^{-1}$ IL-2 for 6 days, were taken as effector cell population. Aliquots of 2000 target cells were mixed with effector cells at effector–target ratios ranging from 100:1 to 12:1 in 96-well round bottom microtitre plates (Greiner, Langenthal, Switzerland). Serial 10-fold dilutions of antibody were added in concentrations ranging from 1 $\mu g\ ml^{-1}$ to 1 ng $ml^{-1}$. The plates were then centrifuged at 800 r.p.m. for 2 min and incubated at 37°C for 4 h. An aliquot of 100 $\mu$l supernatant was counted in an LKB gamma counter (ER, experimental release). Spontaneous release (SR) and maximal release (MR) were assayed by respectively adding medium or 100 $\mu$l of 10% Triton X-100 to the labelled targets. The percentage specific lysis was calculated as $(ER-SR/MR-SR) \times 100\%$.

### Results

### *Generation of chimeric bispecific G250/anti-CD3 MAb*

Chimeric heavy and light chain genes of anti-CD3 MAb were transfected into P3X63Ag8.653 myeloma cells, yielding transfectants producing chimeric anti-CD3 MAb, as was determined by ELISA. The transfectant that had produced the highest amount of antibody (25 $\mu$g per $10^6$ cells) during a 10 day culture, was selected and retransfected with the expression vector containing the chimeric heavy and light chain genes of the G250 MAb (Figure 1). The presence of antibodies with G250 specificity in the culture supernatant of the transfectants was analysed by ELISA using plates coated with anti-idiotype G250 MAb and goat anti-human IgG MAb as detecting antibody as described in Materials and methods. Twenty transfectants that produced antibodies with G250 specificity were selected for analysis of bispecific G250/anti-CD3 MAb production.

The production of chimeric bispecific antibody was analysed by flow cytometry using HPB-ALL T cells, which express the CD3 antigen. HPB-ALL T cells were incubated with culture supernatant of the transfectants followed by the incubation with anti-idiotype G250 MAb (Figure 2a). Control incubations of HPB-ALL T cells with only anti-idiotype G250 MAb revealed that the anti-idiotype G250 MAb did not react with the HPB-ALL cells, and therefore

**Figure 3** Cytotoxicity of cloned human T cells mediated by chimeric bispecific MAb. The human T cell clone, TIL 7.9, was assayed for cytotoxicity against A704 targets in the absence of antibody (□), in the presence of 50 $\mu$l per well culture supernatant containing murine bispecific G250/OKT-3 (▩) or 50 $\mu$l per well culture supernatant containing chimeric bispecific G250/anti-CD3 MAb (■). E/T ratio (effector–target ratio), 2000 targets per well. Results are presented as percentage specific $^{51}$Cr release ± s.d. of triplicate cultures in a 4 h assay. Data shown are representative of three independent experiments. At all E/T ratios the percentage lysis in the presence of antibody differs significantly from the control ($P<0.0001$).

was suitable to detect bispecific antibodies bound to the HPB-ALL T cells. This was confirmed by positive control stainings with murine bispecific G250/OKT-3 (anti-CD3) MAb (Figure 2c). Chimeric G250 MAb did not react with HPB-ALL T cells, as analysed with the anti-idiotype G250 MAb. A low level of cross-reactivity of the anti-idiotype G250 MAb with the anti-CD3 MAb was observed (Figure 2c). Culture supernatant of several transfectants showed an increase in fluorescence as compared with the control incubations of HPB-ALL T cells without culture supernatant, indicating that these transfectants produced antibodies with both G250 and anti-CD3 specificity (Figure 2a). In parallel, HPB-ALL cells, incubated with culture supernatant or control antibodies, were stained with goat anti-human IgG MAb as detecting antibody to determine the total amount of antibodies with anti-CD3 specificity present in the culture supernatant of the transfectants (Figure 2b and d).

Subsequently, the capacity of culture supernatant of the transfectants producing chimeric bispecific cG250/anti-CD3 MAb to trigger activated human $CD8^+$T cells for lysis of target cells that express the G250 antigen was analysed in a cytotoxicity assay. Figure 3 shows the results of supernatant of transfectant T3 no. 36.4. The chimeric bispecific G250/anti-CD3 MAb greatly increased the lysis of A704 target by activated T cells, as was observed for the control murine bispecific G250/OKT-3 (anti-CD3) MAb at effector–target ratios ranging from 50:1 to 6:1.

**Figure 4** Binding of HPLC-purified chimeric bispecific MAb to human T cells and RCC cells. Approximately $5\times10^5$ human T cells of clone TIL 7.9 orA704 renal carcinoma cells were stained with the HPLC fraction corresponding to peak 1 (**c** and **d**) or peak 2 (**e** and **f**, $10\,\mu g\,ml^{-1}$) or PBS (**a** and **b**), followed by an incubation with FITC-labelled goat anti-human IgG and analysed by flow cytometry. (**a**, **c** and **e**) antibody binding to T cells; (**b**, **d** and **f**) antibody binding to A704 cells.

**Figure 5** Cytotoxicity of cloned human T cells mediated by HPLC fractions of chimeric bispecific MAb. HPLC peak 1 (□) and peak 2 (■) of the chimeric bispecific MAb purification were assayed for their capacity to mediate cytotoxicity by the human T cell clone, TIL 7.9, against A704 targets in a 4 h $^{51}$Cr release assay. Antibody was added to the wells at final concentrations of 1, 0.1, 0.01 or 0.001 μg ml$^{-1}$. No antibody was added to the control assays. E/T ratio 25:1, 2000 targets per well. Data shown are representative of three independent experiments. The percentage lysis at 1, 0.1 and 0.01 μg ml$^{-1}$ of peak 2 differs significantly from peak 1 ($P<0.0002$).

*Purification of chimeric bispecific MAb*

Antibody production by transfectant T3 no. 36.4 was considered stable, since 100% of the clones, obtained by limiting dilution cloning of a 4 week culture of the transfectant, produced functional bispecific MAb. The transfectant produced up to 17 μg ml$^{-1}$ of total antibody in spent culture, as measured by ELISA. The percentage of bispecific MAb was approximately 20–25%. This transfectant was chosen for preparative scale antibody purification. All possible antibody variants, resulting from random association of the two types of heavy and light chains produced by the transfectants, were of IgG1 subtype, and therefore cannot be segregated by affinity purification on Protein-A Sepharose, which is based on differences in binding strength of the various subclasses of IgG. Therefore, total chimeric IgG1 was purified from culture supernatant on Protein A Sepharose and subsequently separated into individual antibody fractions by HPLC on Mono S-Sepharose. HPLC analysis of the purified chimeric IgG1 pool produced by transfectant T3 no. 36.4 revealed only two peaks. The position of one peak in the HPLC chromatogram was consistent with that of parental G250 antibody (peak 1). The second peak did not resemble chimeric anti-CD3 MAb and might contain the bispecific antibody fraction (peak 2). To confirm that peak 2 contained bispecific MAb and peak 1 parental G250 MAb, cytotoxicity assays were performed and the binding to G250 antigen-expressing tumour cells and T cells was evaluated.

*Binding of chimeric bispecific MAb to T cells and tumour cells*

The antibody fractions corresponding to the different peaks in the HPLC chromatogram were analysed for binding to T cells and A704 cells expressing the G250 antigen. The human T cell clone, TIL7.9, and A704 cells were incubated with the antibody fractions corresponding with peak 1 or peak 2 respectively, and analysed by flow cytometry. Figure 4 shows that antibody of peak 2 reacted with both A704 cells and T cells, while antibody of peak 1 only bound to A704 cells.

*Cytotoxicity of cloned human T cells mediated by chimeric bispecific MAb*

Both antibody fractions (peaks 1 and 2) of the HPLC separation of the chimeric bispecific MAb were then analysed for their ability to mediate cytotoxicity by human T cells against G250 antigen-positive target cells. The antibody fraction of peak 2 was capable of mediating lysis of A704 target cells, whereas peak 1 antibody was not (Figure 5). Induction of lysis mediated by peak 2 (cG250/anti-CD3) was still detectable at an antibody concentration of 0.01 μg ml$^{-1}$ (Figure 5), which shows that bispecific antibody-mediated cytotoxicity is effective at low doses of antibody.

Tumour cell lines that did not express the G250 antigen were not lysed in the presence of the bispecific antibody. The chimeric bispecific MAb mediated lysis of target cells with a relatively low expression of the G250 antigen as well as targets with a higher expression of the G250 antigen (Figure 6). The efficiency of lysis of G250-expressing cells, induced by chimeric bispecific MAb, was dependent on the concentration of the bispecific MAb as well as on the target cell line used.

As low levels of G250 expression on T cells might lead to cytokine release and autokill of T cells by the chimeric bispecific G250/anti-CD3 MAb, we have tested TNF-α release by T cells in the presence of the chimeric bispecific MAb, using the WEHI assay (Luiten *et al.*, 1996). No TNF-α release was seen at concentrations of 0.1 μg ml$^{-1}$ bispecific MAb or parental G250 MAb, whereas chimeric anti-CD3 MAb did lead to TNF-α release.

Based on the binding specificities of both antibody peaks and the difference in capacity to induce cytotoxicity by T cells, peak 2 was considered to contain chimeric bispecific G250/anti-CD3 MAb.

*Analysis of mRNA expression for antibody heavy and light chains*

In the chromatogram of the HPLC separation of the chimeric bispecific antibody fractions a third peak, representing the parental anti-CD3 MAb, was absent. This might indicate that one of the chains for the anti-CD3 MAb was not produced by clone T3 no. 36.4, inhibiting the production of parental anti-CD3. Therefore, the presence of anti-CD3 andG250 MAb heavy and light chain mRNA in the chimeric bispecific MAb-producing transfectant, T3 no. 36.4, was determined by RT–PCR using primer sets specific for the variable regions of the G250 or the anti-CD3 MAb heavy and light chains. The parental cell line and cell lines producing either chimeric anti-CD3 MAb or chimeric G250 MAb were used as control cell lines to check the specificity of the primers. Figure 7 shows that the primer sets specific for the variable regions of anti-CD3 MAb heavy and light chains generated a PCR product of mRNA derived from the anti-CD3 MAb-producing cell line as well as of mRNA derived from the chimeric bispecific-producing clone, T3 no. 36.4, indicating that the transfectant contains mRNA for both anti-CD3 antibody chains. In the control experiment these primer sets did not yield a PCR product with the G250 MAb-producing cell line not containing anti-CD3 MAb mRNA. Analogous results were obtained in analyses for the G250 antibody mRNA. From both the G250 MAb-producing cell line and the clone T3 no. 36.4 a PCR product was obtained with primer sets specific for either the heavy or light chain variable regions of G250 MAb, and not from the anti-CD3 MAb-producing cell line. Clone T3 no. 36.4 appeared to contain mRNA for heavy and light chains of both the anti-CD3 and the G250 MAb, indicating that all chains transfected were expressed in clone T3 no. 36.4.

*Cytotoxicity of human PBLs mediated by the chimeric bispecific MAb*

Bispecific antibody-mediated cytotoxicity *in vivo* will deal with lymphocytes of various phenotypes as effector cell population.

**d**

| *Target cell line* | *G250 antigen expression*[a] |
|---|---|
| A704 | 120 |
| SK-RC-1 | 47 |
| SK-RC-7 | 26 |
| SK-RC-45 | 0 |
| ACHN | 0 |

**Figure 6** Lysis of RCC cell lines by cloned human T cells in the presence of chimeric bispecific MAb. The RCC cell lines, A704, SK-RC-1, SK-RC-7, SK-RC-45 and ACHN were stained for G250 expression and analysed by flow cytometry (**a** and **b**). TIL7.9 cells were assayed for cytotoxicity against A704 (○), SK-RC-1 (●), SK-RC-7 (+), SK-RC-45 (△) and ACHN (▲) in the presence of 1, 0.1, 0.01, 0.001 or 0 μg ml$^{-1}$ chimeric bispecific G250/anti-CD3 MAb, E/T ratio 25:1, 2000 targets per well (**c**). **d**, G250 antigen expression of RCC cell lines.[a] Mean channel number (MCN) after subtraction of autofluorescence.

To show that our chimeric bispecific antibody is capable of inducing lysis by a heterogenous population of lymphocytes, peripheral blood lymphocytes (PBLs) of different donors were used as effector cells. Since chimeric bispecific MAb was unable to induce cytotoxicity with unstimulated PBLs or with PBLs after an overnight stimulation with 100 U ml$^{-1}$ IL-2 (van Ravenswaay Claasen *et al.*, 1993), PBLs were cultured with 100 U ml$^{-1}$ IL-2 for 6 days before cytotoxicity assays. Figure 8 shows that the chimeric bispecific G250/anti-CD3 MAb induced significant lysis of A704 targets with PBLs from donor A or donor C compared with controls without antibody, whereas PBLs from donor B showed a marginal enhancement of lysis. Parental chimeric anti-CD3 MAb and G250 MAb do not mediate cytotoxicity of activated PBLs at the concentration of 1 μg ml$^{-1}$ (data not shown). Activation of PBLs from donor A, but not donor B, enhanced the level of lysis of SK-RC-7 target cells mediated by chimeric bispecific G250/anti-CD3 MAb (data not shown). The amount of lytic activity that was induced in a 4 h incubation varied between donors (van Ravenswaay Claasen *et al.*, 1993). This might be owing to differences in the percentage of $CD8^+$ cells among the IL-2-stimulated PBLs. Flow cytometric analysis revealed that PBLs from donor A indeed contained more $CD8^+$T cells (45%) than PBLs from donor B (24%). These results show that IL-2-activated PBLs can be triggered to lyse G250 antigen-positive RCC target cells in the presence of bispecific cG250/anti-CD3 MAb.

### Discussion

Renal cell carcinoma has been studied intensively to evaluate different approaches to immunotherapy. Phase I and II clinical trials in which patients with RCC were treated with IL-2 revealed the susceptibility of RCC to IL-2, but also showed the toxicity of this cytokine when administered systemically in high doses. IL-2-based therapy is therefore limited by maximum tolerated dose (Gaynor *et al.*, 1990). The response rates between these trials varied between 12 and 30% (Marumo *et al.*, 1989; Hayat *et al.*, 1991; Jensen *et al.*, 1990; Whitehead *et al.*, 1990; Rosenberg *et al.*, 1989), but occasionally no responses were observed (Abrams *et al.*, 1990). Co-administration of lymphokine-activated killer (LAK) cells (Parkinson *et al.*, 1990; Fisher *et al.*, 1988; Foon *et al.*, 1992; Palmer *et al.*, 1992) or tumour-infiltrating lymphocytes (TILs) from RCC (Bukowski *et al.*, 1991) did not result in a significantly better response rate. Several other cytokines, such as interferon (IFN)-α (Muss *et al.*, 1987; Feruglio *et al.*, 1992), IFN-β and IFN-γ (Ernstoff *et al.*, 1992), have been studied for anti-tumour effects in RCC patients. Clinical data of IFN-β treatment combined with IL-2 suggest a better response rate than obtained with IFN-β or IL-2 therapy alone (Krigel *et al.*, 1990). These clinical data show that RCCs are responsive to immunotherapy, but that the anti-tumour effects of cytokines are limited by the toxicity of the therapy used.

Directing effector cells towards the tumour by using bispecific antibodies is a method to increase the specificity of the effector cell population for the targeted tumour cells. Such bispecific MAbs have been demonstrated to induce effective lysis of tumour cells *in vitro*. Limited patient studies showed localisation of bispecific antibody-targeted effector cells to the tumour and cross-linking of effector cells with tumour cells (Kroesen *et al.*, 1993). A study of intraperitoneal treatment of ovarian carcinoma with the bispecific OC/TR MAb which recognises both the carcinoma-associated antigen

**Figure 7** Analysis of antibody heavy and light chain mRNA of the anti-CD3 and G250 MAb. RT–PCR reactions using primer sets specific for the anti-CD3 and G250 MAb heavy (HC) and light chains (LC) were performed on mRNA isolated from (1) the untransfected cell line, P3X63Ag8.653 (2); the cell line 4B5 producing chimeric anti-CD3 MAb (3); chG250 7C1 producing chimeric G250 MAb; and (4) the transfectant T3 no. 36.4. The pictures show an ethidium bromide staining of the PCR products electrophoresed on a 2% agarose gel.

**Figure 8** Cytotoxicity of IL-2-activated human PBL mediated by chimeric bispecific MAb. PBLs from three donors, preactivated with 100 U ml$^{-1}$ IL-2 for 6 days, were assayed for cytotoxicity against the RCC cell line A704 in the presence or absence of 1 $\mu$g ml$^{-1}$ purified chimeric bispecific G250/anti-CD3 MAb. At 2000 targets per well, (□) E/T ratio 100:1, without MAb; (▨) E/T ratio 50:1, without MAb; (▩) E/T ratio 100:1, with MAb; (■) E/T ratio 50:1, with MAb. Data shown are representative of three independent experiments. The percentage lysis observed for donor A ($P<0.0001$) and C ($P<0.02$) in the presence of MAb differ significantly from the controls. Donor B (E/T ratio 50:1), $P<0.02$.

MOv18 expressed on ovarian carcinoma and the CD3 complex on T cells, resulted in several cases of tumour regression (Bolhuis *et al.*, 1992; Canevari *et al.*, 1995).

The G250 antigen is an attractive target for antibody-mediated therapy, because of its nearly exclusive expression in renal carcinoma cells. Studies with the G250 MAb and the murine bispecific G250/OKT-3 (anti-CD3) MAb have shown preferential localisation of these antibodies to the tumour (Oosterwijk *et al.*, 1993; van Dijk *et al.*, 1991) and anti-tumour effects in RCC-xenografted nude mice (van Dijk *et al.*, 1994). The G250/anti-CD3 MAb was chimerised to decrease immunogenicity of the bispecific antibody in patients, because studies in patients with murine anti-tumour antibodies and bispecific antibodies are limited by the development of HAMA responses. These limitations may be greatly reduced by using chimeric antibodies for therapy (LoBuglio *et al.*, 1989; Meredith *et al.*, 1991). A chimeric bispecific G250/anti-CD3 MAb provides the possibility of performing patient studies, in which multiple doses of the bispecific antibody can be administered over a long treatment period hopefully resulting in more extensive tumour regression.

We generated a cell line that produces chimeric bispecific G250/anti-CD3 MAb stably by supertransfection of a cell line producing chimeric anti-CD3 MAb with a single expression vector encoding both chimeric heavy and light chains of the G250 MAb. The chimeric bispecific G250/anti-CD3 MAb produced by the supertransfected cells, was demonstrated to bind both to G250-expressing RCC cells and to human T cells. Chimeric bispecific G250/anti-CD3 MAb is capable of mediating lysis of G250 antigen-positive RCC cells by cloned human CD8$^+$T cells at concentrations as low as 0.01 $\mu$g ml$^{-1}$. Tumour cells with a relatively low expression of the G250 antigen were shown to be lysed equally as well as tumour cells with a high G250 antigen expression.

The chromatogram of the HPLC analysis of the antibody pool produced by clone T3 no. 36.4 revealed only two sharp and symmetrical peaks. One peak (1) corresponded to the chromatogram of G250 MAb and the other peak (2) represented the bispecific antibody fraction. The absence of a third peak representing parental anti-CD3 MAb was not the result of the loss of either the anti-CD3 MAb heavy or light chain, since RT–PCR analysis showed the presence of mRNA for all four antibody chains. The chromatographic separation method has been used previously to purify bispecific OVTL3/OKT-3 MAb (van Ravenswaay Claasen *et al.*, 1993), murine bispecific OC/TR (Warnaar *et al.*, 1994), chimeric bispecific OC/TR (Coney *et al.*, 1996) and murine bispecific G250/OKT-3 MAb (van Dijk *et al.*, 1989), and was shown to separate all combinations of heavy and light chains into distinguishable peaks. Therefore, we expect peak 2 of the chimeric G250/anti-CD3 MAb separation to contain IgG molecules composed of the parental heavy and light chains. We have no data to exclude mismatches in peak 2. However, De Lau *et al.* (1991) have shown that the production of two types of heavy and light chains by a quadroma does not necessarily lead to random association of these chains. Depending on the combination of parental antibodies, preferential association of heavy and light chains may occur, resulting in the absence of some heavy and light chain combinations. In addition, the profiles of the chromatograms of different clones producing the same bispecific OC/TR MAb have been shown to differ greatly, as a result of different rates of synthesis of the antibody chains (Warnaar *et al.*, 1994). In both the murine OC/TR-producing hybridoma and the chimeric OC/TR-producing hybridoma, clones were found that produced only one parental antibody and the bispecific MAb. The chromatogram of clone T3 no. 36.4 therefore represents one of the variants of the chromatograms that were observed among different clones producing other bispecific MAbs.

For any RCC patient to be treated with chimeric bispecific

G250/anti-CD3 MAb, the effector cell population to be activated will be a T cell of variable phenotype residing in the PBLs. IL-2-activated PBLs can be activated *in vitro* by chimeric bispecific G250/anti-CD3 MAb to lyse G250 antigen-expressing RCC cells. The activated PBL cytotoxicity may vary between donors and may vary according to the activation protocol (van Ravenswaay Claasen *et al.*, 1993). The *in vitro* cytotoxicity data suggest that treatment of RCC patients with chimeric bispecific G250/anti-CD3 MAb requires preactivation of the patients PBLs. Activated PBLs can be administered to the patient in combination with chimeric bispecific G250/anti-CD3 MAb, or alternatively IL-2 can be given as in current IL-2-based regimens and bispecific G250/anti-CD3 MAb can be co-administered to enhance the effectiveness of IL-2-based therapy.

The murine versions of G250 and anti-CD3 MAb were immunogenic in patients, which was not the case for the chimeric versions (Dr E Oosterwijk, personal communication, Coney *et al.*, 1996; Oosterwijk *et al.*, 1993; Canevari et al., 1995). Since the components of the chimeric bispecific G250/anti-CD3 MAb did not induce an immune response, it is reasonable to expect that the chimeric bispecific MAb will also be non-immunogenic. Although humanisation of antibodies through CDR grafting is generally assumed to reduce immunogenicity further compared with chimeric antibodies, there is no solid patient data to support this notion. Humanised and chimeric antibodies have reduced immunogenicity compared with murine antibodies, however no direct comparison between humanised and chimeric MAbs has been performed. The fact that chimerisation of the G250 and anti-CD3 MAbs apparently abrogates the patients' immune response to the infused antibody, makes humanisation of the antibodies unnecessary. It is likely that any residual immunogenicity of chimeric or humanised antibodies will be idiotype dependent.

In conclusion, the chimeric bispecific G250/anti-CD3 MAb may represent a useful tool for enhancing the results obtained in RCC treatment using IL-2.

**Abbreviations**

MAb, monoclonal antibody; RCC, renal cell carcinoma; HAMA, human anti-mouse antibody; $V_H$, variable region of antibody heavy chain; $V_K$, variable region of antibody kappa chain; $C_H$, constant region of antibody heavy chain; $C_K$, constant region of antibody kappa chain; *gpt*, xanthine guanine phosphoribosyl transferase; *neo*, neomycin; RT-PCR, reverse transcriptase-polymerase chain reaction.

**Acknowledgements**

We thank Jaap van Eendenburg, Inge Briaire, Hellen Bakker, Coby Huiskens, Jack de Graaf, Toin Backhaus, Jim Mackle and David Sanborn for technical assistance. This research was supported by the Dutch Kidney Foundation, The Netherlands (grant C92.1264).

### References

ABRAMS JS, RAYNER AA, WIERNIK PH, PARKINSON DR, EISENBERGER M, ARONSON FR, GUCALP R, ATKINS MB AND HAWKINS MJ. (1990). High-dose recombinant interleukin-2 alone: a regimen with limited activity in the treatment of advanced renal cell carcinoma. *J. Natl Cancer Inst.*, **82,** 1202-1206.

BERG J, LOTSCHER E, STEIMER KS, CAPON DJ, BAENZIGER J, JACK HM AND WABL M. (1991). Bispecific antibodies that mediate killing of cells infected with human immunodeficiency virus of any strain. *Proc. Natl Acad. USA*, **88,** 4723-4727.

BOLHUIS RL, LAMERS CH, GOEY SH, EGGERMONT AM, TRIMBOS JB, STOTER G, LANZAVECCHIA A, DI RE E, MIOTTI S, RASPAGLIESI F, RIVOLTINI L AND COLNAGHI MI. (1992). Adoptive immunotherapy of ovarian carcinoma with bs-MAb-targeted lymphocytes: a multicenter study. *Int. J. Cancer* (suppl.), **7,** 78-81.

BRISSINCK J, DEMANET C, MOSER M, LEO O AND THIELEMANS K. (1991). Treatment of mice bearing bcll lymphoma with bispecific antibodies. *J. Immulol.*, **147,** 4019-4026.

BUKOWSKI RM, SHARFMAN W, MURTHY S, RAYMAN P, TUBBS R, ALEXANDER J, BUDD GT, SERGI JS, BAUER L, GIBSON V, STANLEY J, BOYETT J, PONTES E AND FINKE JH. (1991). Clinical results and characterization of tumor-infiltrating lymphocytes with or without recombinant interleukin 2 in human metastatic renal cell carcinoma. *Cancer Res.*, **51,** 4199-4205.

CANEVARI S, STOTER G, ARIENTI F, BOLIS G, COLNAGHI MI, DIRE EM, EGGERMONT AM, GOEY SH, GRATAMA JW, LAMERS CH, NOOY MA, PARMIANI G, RASPAGLIESI F, RAVAGNANI F, SCARFONE G, TRIMBOS JB, WARNAAR SO AND BOLHUIS RLH. (1995). Regression of advanced ovarian carcinoma by intraperitoneal treatment with autologous T lymphocytes retargeted by a bispecific monoclonal antibody. *J. Natl Cancer Inst.*, **87,** 1463-1469.

CONEY LR, SANBORN D, LAMERS CHL, ZURAWSKI VC Jr AND WARNAAR SO. (1996). A chimeric bispecific antibody that exhibits potent cytolysis of tumor cell lines expressing the tumor-associated folate receptor and decreased immunogenicity in patients (*submitted for publication*).

COURTNAY-LUCK NS, EPENETOS AA, MOORE R, LARCHE M, PECTASIDES D, DHOKIA B AND RITTER MA. (1986). Development of primary and secondary immune responses to mouse monoclonal antibodies used in the diagnosis and therapy of malignant neoplasms. *Cancer Res.*, **46,** 6489-6493.

DE LAU WBM, HEIJE K, NEEFJES JJ, OOSTERWEGEL M, ROZEMULLER E AND BAST BJEG. (1991). Absence of preferential homologous H/L chain association in hybrid hybridomas. *J. Immunol.*, **146,** 906-914.

DIJK VAN J, OOSTERWIJK E, KROONENBURGH VJPG, JONAS U, FLEUREN GJ, PAUWELS EKJ AND WARNAAR SO. (1988). Perfusion of tumor-bearing kidneys as a model for scintigraphic screening of monoclonal antibodies. *J. Nucl. Med.*, **29,** 1078-1082.

DIJK VAN J, WARNAAR SO, EENDENBURG VDH, THIENPONT M, BRAAKMAN E, BOOT JHA, FLEUREN GJ AND BOLHUIS RLH. (1989). Induction of tumor-cell lysis by bi-specific monoclonal antibodies recognizing renal-cell carcinoma and CD3 antigen. *Int. J. Cancer*, **43,** 344-349.

DIJK VAN J, ZEGVELD ST, FLEUREN GJ AND WARNAAR SO. (1991). Localization of monoclonal antibody G250 and bispecific monoclonal antibody CD3/G250 in human renal-cell carcinoma xenografts: relative effects of and size affinity. *Int. J. Cancer*, **48,** 738-743.

DIJK VAN J, UEMURA H, BENIERS AJMC, PEELEN WP, ZEGVELD ST, FLEUREN GJ, WARNAAR SO AND OOSTERWIJK E. (1994). Therapeutic effects of monoclonal antibody G250, interferons and tumor necrosis factor, in mice with renal-cell carcinoma xenografts. *Int. J. Cancer*, **56,** 262-268.

ERNSTOFF MS, GOODING W, NAIR S, BAHNSON RR, MIKETIC LM, BANNER B, DAY R, WHITESIDE T, TITUS-ERNSTOFF L AND KIRKWOOD JM. (1992). Immunological effects of treatment with sequential administration of recombinant interferon gamma and $\alpha$ in patients with metastatic renal cell carcinoma during a phase I trial. *Cancer Res.*, **52,** 851-856.

FANGER MW, SEGAL DM AND WUNDERLICH JR. (1990). Going both ways: bispecific antibodies and targeted cellular cytotoxicity. *Science*, **4,** 2846-2849.

FERUGLIO C, ZAMBELLO R, TRENTIN L, BULIAN P, FRANCESCHI T, CETTO GL AND SEMENZATO G. (1992). Cytotoxic *in vitro* function in patients with metastatic renal cell carcinoma before and after alpha-2b-interferon therapy: effects of activation with recombinant interleukin-2. *Cancer*, **69,** 2525-2531.

FISHER RI, COLTMAN CA, DOROSHOW JH, RAYNER AA, HAWKINS MJ, MIER JW, WIERNIK P, MCMANNIS JD, WEISS GR, MARGOLIN KA, GEMLO BT, HOTH DF, PARKINSON DR AND PAIETTA E. (1988). Metastatic renal cancer treated with interleukin-2 and lymphokine-activated killer cells. *Ann. Intern. Med.*, **108,** 518-523.

FOON KA, WALTHER PJ, BERNSTEIN ZP, VAICKUS L, RAHMAN R, WATANABE H, SWEENEY J, PARK J, VESPER D, RUSSELL D, WALKER RA, DARROW TL, JUHANI LINNA T, FARMER DL, LYNCH WJ JR, HUBEN R AND GOLDROSEN MH. (1992). Renal cell carcinoma treated with continuous-infusion interleukin-2 with *ex vivo*-activated killer cells. *J. Immunother.*, **11,** 184-190.

GARRIDO MA, VALDAYO MJ, WINKLER DF, TITUS JA, HECHT TT, PEREZ P, SEGAL DM AND WUNDERLICH JR. (1990). Targeting human T-lymphocytes with bispecific antibodies to react against human ovarian carcinoma cells growing in nu/nu mice. *Cancer Res.*, **50**, 4227–4232.

GAYNOR ER, WEISS GR, MARGOLIN KA, ARONSON FR, SZNOL M, DEMCHAK P, GRIMA KM, FISHER RI, BOLDT DH, DOROSHOW JH, BAR MH, HAWKINS MJ, MIER JW AND CALIENDO G. (1990). Phase I study of high-dose continuous-infusion recombinant interleukin-2 and autologous lymphokine-activated killer cells in patients with metastatic or unresectable malignant melanoma and renal cell carcinoma. *J. Natl Cancer Inst.*, **82**, 1397–1402.

HAYAT K, RODGERS S, BRUCE L, REES RC, CHAPMAN K, REEDER S, DORREEN MS, SHERIDAN E, SREENIVASAN T AND HANCOCK BW. (1991). Malignant melanoma and renal cell carcinoma: immunological and haematological effects of recombinant human interleukin-2. *Eur. J. Cancer*, **27**, 1009–1014.

JENSEN PB AND BIRCH C. (1990). Low-dose regimen of interleukin-2 for metastatic renal carcinoma. *Lancet*, **335**, 1522–1523.

KRIGEL RL, PADAVIC-SHALLER KA, RUDOLPH AR, KONRAD M, BRADLEY EC AND COMIS RL. (1990). Renal cell carcinoma: treatment with recombinant interleukin-2 plus beta-interferon. *J. Clin. Oncol.*, **8**, 460–467.

KROESEN BJ, TER HAAR A, SPAKMAN H, WILLEMSE P, SLEIJFER DT, DE VRIES EGE, MULDER NH, BERENDSEN HH, LIMBURG PC, THE TH AND DE LEIJ L. (1993). Local antitumour treatment in carcinoma patients with bispecific-monoclonal-antibody-redirected T cells. *Cancer Immunol. Immunother.*, **37**, 400–407.

LOBUGLIO AF, WHEELER RH, TRANG J, HAYNES A, ROGERS K, HARVEY EB, SUN L, GRAYEB J AND KHAZAELI MB. (1989). Mouse/human chimeric monoclonal antibody in man: kinetics and immune response. *Proc. Natl Acad. USA*, **86**, 4220–4224.

LUITEN RM, FLEUREN GJ, WARNAAR SO AND LITVINOV SV. (1996). Target-specific activation of mast cells by immunoglobulin E reactive with a renal-cell-carcinoma-associated antigen. *Lab. Invest.*, **74**, 467–475.

MARUMO K, BABA S, MURAKI J, JITSUKAWA S, UENO M, HATA M, TACHIBANA M, TAZAKI H AND DEGUCHI N. (1989). Immunologic study of human recombinant interleukin-2 (low dose) in patients with advanced renal cell carcinoma. *Urology*, **33**, 219–225.

MEREDITH RF, LOBUGLIO AF, PLOTT WE, ORR RA, BREZOVICH IA, RUSSELL CD, HARVEY EB, YESTER MV, WAGNER AJ, SPENCER SA, WHEELER RH, SALEH MN, ROGERS KJ, POLANSKY A, SALTER MM AND KHAZAELI MB. (1991). Pharmacokinetics, immune response, and biodistribution of iodine-131-labeled chimeric mouse/human IgG1,k 17-1A monoclonal antibody. *J. Nucl. Med.*, **32**, 1162–1168.

MEZZANZANICA D, CANEVARI S, MENARD S, PUPA SM, TAGLIABUE E, LANZAVECCHIA A AND COLNAGHI MI. (1988). Human ovarian carcinoma lysis by cytotoxic T cells targeted bybi-specific monoclonal antibodies: analysis of the antibody components. *Int. J. Cancer*, **41**, 609–615.

MUSS HB, COSTANZI JJ, LEAVITT R, WILLIAMS RD, KEMPF RA, POLLARD R, OZER H, ZEKAN PJ, GRUNBERG SM, MITCHEL MS, CAPONERA M, GAVIGAN M, ERNEST ML, VENTURI C, GREINER JW AND SPIEGEL RJ. (1987). Recombinant alpha interferon: a randomized trial of two routes of administration. *J. Clin. Oncol.*, **5**, 286–291.

NISTICO P, MORTARINI R, DEMONTE LB, MAZZOCCHI A, MARIANI M, MALAVASI F, PARMIANI G, NATALI PG AND ANICHINI A. (1992). Cell retargeting by bispecific monoclonal antibodies. Evidence of bypass of intratumor susceptibility to lysis in human melanoma. *J. Clin. Invest.*, **90**, 1093–1099.

OOSTERWIJK E, RUITER DJ, HOEDEMAEKER PJ, PAUWELS EKJ, JONAS U, ZWARTENDIJK J AND WARNAAR SO. (1986). Monoclonal antibody G250 recognizes a determinant present in renal-cell carcinoma and absent from normal kidney. *Int. J. Cancer*, **38**, 489–494.

OOSTERWIJK E, BANDER NH, DIVGI CR, WELT S, WAKKA JC, FINN RD, CARSWELL EA, LARSON SM, WARNAAR SO, FLEUREN GJ, OETTGEN HF AND OLD LJ. (1993). Antibody localization in human renal cell carcinoma: a phase I study of monoclonal antibody G250. *J. Clin. Oncol.*, **11**, 738–750.

PALMER PA, VINKE J, EVERS P, POURREAU C, OSKAM R, ROEST G, VLEMS F, BECKER L, LORIAUX E AND FRANKS CR. (1992). Continuous infusion of recombinant interleukin-2 with or without autologous lymphokine activated killer cells for the treatment of advanced renal cell carcinoma. *Eur. J. Cancer*, **28A**, 1038–1044.

PARKINSON DR, FISHER RI, RAYNER AA, PAIETTA E, MARGOLIN KA, WEISS GR, MIER JW, SZNOL M, GAYNOR ER, BAR MH, GUCALP R, BOLDT DH, MILLS B AND HAWKINS MJ. (1990). Therapy of renal cell carcinoma with interleukin-2 and lympholine-activated killer cells: phase II experience with a hybrid bolus and continuous infusion interleukin-2 regimen. *J. Clin. Oncol.*, **8**, 1630–1636.

QIAN JH, TITUS JA, ANDREW SM, MEZZANZANICA D, GARRIDO MA, WUNDERLICH JR AND SEGAL DM. (1991). Human peripheral blood lymphocytes targeted with bispecific antibodies release cytokines that are essential for inhibiting tumor growth. *J. Immunol.*, **146**, 3250–3256.

RAVENSWAAY CLAASEN VAN HH, GRIENDT VJ, MEZZANZANICA D, BOLHUIS RLH, WARNAAR SO AND FLEUREN GJ. (1993). Analysis of production, purification, and cytolytic potential of bispecific antibodies reactive with ovarian-carcinoma-associated antigens and the T-cell antigen CD3. *Int. J. Cancer*, **55**, 128–136.

RIVA P, TISON V, ARISTA A, STURIALE C, FRANCESCHI G, RIVA N, CASI M, MOSCATELLI G, CAMPORI F AND SPINELLI A. (1993). Radioimmunotherapy of gastrointestinal cancer and glioblastomas. *Int. J. Biol. Markers*, **8**, 192–197.

ROSENBERG SA, LOTZE MT, YANG JC, AEBERSOLD PM, LINEHAN WM, SEIPP CA AND WHITE DE. (1989). Experience with the use of high-dose interleukin-2 in the treatment of 652 cancer patients. *Ann. Surg.*, **210**, 474–484.

SALEH MN, KHAZAELI MB, GRIZZLE WE, WHEELER RH, LAWSON S, LIU T, RUSSELL C, MEREDITH R, SCHLOM J AND LOBUGLIO AF. (1993). A phase I clinical trial of murine monoclonal antibody D612 in patients with metastatic gastrointestinal cancer. *Cancer Res.*, **53**, 4555–4562.

SCHROFF RW, FOO KH, BEATTY SM, OLDHAM RK AND MORGAN AC. (1985). Human anti-murine immunoglobulin responses in patients receiving monoclonal antibody therapy. *Cancer Res.*, **45**, 879.

SHAWLER DL, BARTHOLOMEW RM, SMITH LM AND DILLMAN RO. (1985). Human immune response to multiple injections of murine monoclonal IgG. *J. Immunol.*, **135**, 1530.

TIBBEN JG, BOERMAN OC, CLAESSENS RA, CORSTENS FH, VAN DEUREN M, DE MULDER PH, VAN DER MEER JW, KEIJSER KG AND MASSUGER LF. (1993). Cytokine release in an ovarian carcinoma patient following intravenous administration of bispecific antibody OC/TR $F(ab)_2$. *J. Natl Cancer. Inst.*, **85**, 1003–1004.

WARNAAR SO, PAUS DE V, LARDENOIJE R, MACHIELSE BNM, GRAAF D, BREGONJE M AND HAARLEM V. (1994). Purification of bispecific $F(ab')_2$ from murine trinoma OC/TR with specificity for CD3 and ovarian cancer. *Hybridoma*, **13**, 519–525.

WHITEHEAD RP, WARD D, HEMINGWAY L, HEMSTREET GP, III, BRADLEY E AND KONRAD M. (1990). Subcutaneous recombinant interleukin 2 in a dose escalating regimen in patients with metastatic renal cell adenocarcinoma. *Cancer Res.*, **50**, 6708–6715.